# Associations between food and beverage purchases and skin carotenoids among diverse small food retail store customers

**DOI:** 10.1017/S1368980023001581

**Published:** 2023-11

**Authors:** Jocelyn Dixon, Virginia C Stage, Kimberly P Truesdale, Qiang Wu, Kathryn Kolasa, Lindsey Haynes-Maslow, Jared T McGuirt, Stephanie Jilcott Pitts

**Affiliations:** 1 Departments of Nutrition Science and Public Health, East Carolina University, Greenville, USA; 2 North Carolina State University, Raleigh, NC, USA; 3 Department of Nutrition, UNC-Chapel Hill, Chapel Hill, USA; 4 Department of Public Health, ECU, Greenville 27834, USA; 5 Department of Family Medicine, ECU, Greenville, USA; 6 Department of Health Policy and Management, UNC-Chapel Hill, Chapel Hill, USA; 7 Department of Nutrition, UNC-Greensboro, Greensboro, USA

**Keywords:** Skin carotenoids, Healthy purchases, Corner stores, Fruits and vegetables

## Abstract

**Objective:**

To determine if customer purchases at small food stores are associated with healthfulness of the diet as approximated by skin carotenoids.

**Design:**

This is a cross-sectional survey of customers in small food stores regarding demographics and food purchases. Food and beverage purchases were classified as ‘healthy’ or ‘non-healthy’ and ‘carotenoid’ *v*. ‘non-carotenoid’ using a systematic classification scheme. Fruit and vegetable intake was objectively assessed using a non-invasive device to measure skin carotenoids. Associations between variables of interest were examined using Pearson’s correlation coefficients, *t* tests and multiple linear regression analyses.

**Setting:**

Twenty-two small food retail stores in rural (*n* 7 stores) and urban (*n* 15) areas of North Carolina.

**Participants:**

Customers of small food stores

**Results:**

Of study participants (*n* 1086), 55·1 % were male, 60·0 % were African American/Black and 4·2 % were Hispanic, with a mean age of 43·5 years. Overall, 36 % purchased at least one healthy item, and 7·6 % of participants purchased a carotenoid-containing food/beverage. Healthy foods and beverages purchased included produce, lean meats, 100 % juices, plain popcorn, plain nuts, milk and yogurt. Unhealthy items included non-100 % juices, crackers, chips, candy, cakes and donuts. Purchase of a healthy or carotenoid-containing item was positively associated with skin carotenoid scores (*P* = 0·002 and 0·006, respectively).

**Conclusions:**

A relatively small proportion of customers purchased any healthy or carotenoid-containing foods and beverages, and those who did purchase healthy options had higher skin carotenoid scores. Future research should confirm these findings in different populations.

In rural and underserved communities, small food retailers (e.g. corner and convenience stores) may be one of the only food retailers available^(1,2)^. Small food retailers typically sell foods and beverages that are less healthy when compared to large food retailers, which perpetuates the cycle of low access and poor dietary quality among rural and disadvantaged communities^(3,4)^. To improve access to healthy foods, public health interventions have been implemented to stock and promote healthier foods in small food retailers^(5–8)^. More research is needed to better understand how food and beverage purchases at these small food retailers are associated with dietary intake^(5,9)^.

One key component of a healthy diet is fruit and vegetable (FV) intake. Traditional methods to assess FV intake include self-reported measures. Unfortunately, these measures are often fraught with social desirability bias^(10–12)^. Carotenoids are the colourful phytonutrients found in vegetables and fruits, while plasma carotenoids are correlated with FV intake and serve to approximate overall FV intake^(13,14)^. Measurement of skin carotenoid score (SCS) is emerging as a promising method to assess FV intake in field-based nutrition studies, as it is relatively quick and portable^(15,16)^. To date, no studies examine healthfulness of purchases at small food retailers and associations with skin carotenoids. Therefore, the purpose of this study was to determine if customer purchases at small food stores are associated with the overall healthfulness of dietary intake as approximated by SCS.

## Methods

### Research design and setting

This cross-sectional study is a secondary analysis of data from a larger evaluation study of the North Carolina Healthy Food Small Retailer Program (NC HFSRP), which is detailed in other manuscripts^(5,9,17–20)^. A total of twenty-two small food retailers were evaluated, including customer intercept surveys and bag checks among 1261 customers between 2017 and 2020. While the NC HFSRP was first designed to promote healthy food access in rural areas, it was eventually expanded so that seven of the stores were in rural areas and fifteen were in more urban areas. To classify stores as rural or urban, the stores were spatially joined to census tract-level data from the USDA Food Access Research Atlas dataset which defines rural and urban based on the geographic centroid of the census tract, with census tracts containing more than 2500 people being urban, and all others being rural^(21)^.

### Study population and data collection

Participants in this study were a convenience sample of adults (18 years or older) who purchased food or beverage items at small food retailers participating in the larger evaluation study^(18)^. Verbal consent was given after research staff provided individuals with information about the study. If interested and eligible, participants completed a survey with demographic information and researchers conducted a bag check by recording detailed information on each item purchased, consistent with procedures in prior studies^(22,23)^.

### Skin carotenoid scores

SCS were measured using the Veggie Meter®, a non-invasive, portable device that uses pressure-mediated reflection spectroscopy to quantify skin carotenoids^(24)^. The Veggie Meter® has been validated against plasma carotenoids and self-reported FV intake and has been successfully used in community settings among racially and ethnically diverse participants^(18,25–28)^. SCS are associated with plasma carotenoids and FV intake^(29,30)^, key components of the federal Dietary Guidelines for Americans^(31–34)^.

Each participant’s right index finger was inserted into the instrument’s finger cradle to bring the fingertip in contact with the collecting contact lens, while simultaneously applying light pressure so that blood was temporarily squeezed out of the measured skin region^(27)^. Participants’ fingers were scanned three times in the single-scan mode to obtain a mean SCS. SCS range from 0 to 800, with a higher score indicating more skin carotenoids present. The device was calibrated with a light and dark reference before each measurement session.

### Bag check purchase data

Bag check data were analysed to generate a total of four scores detailing individuals’ purchases: (1) Healthy Purchases; (2) Healthy Purchases-Proportion; (3) Carotenoid-Containing Purchases; and (4) Carotenoid Containing Purchases-Proportion. To classify foods, one Registered Dietitian/Nutritionist (RDN (PhD) and one Nutritional Epidemiologist (PhD) were given a list of all recorded food and beverage items and independently classified items as healthy or unhealthy and carotenoid-containing or non-carotenoid-containing. Coding results were compared, and a second RDN, PhD, served as the tiebreaker on items when consensus was not reached. The criteria used to classify healthy items followed the 2020 Dietary Guidelines for Americans and included fruits, vegetables, eggs, beans, bread, low-fat dairy products, lean protein and 100 % juice^(31)^. Common non-healthy items included processed meats, sweetened drinks, salty and sweet snacks, and ready-to-eat meals^(30,34)^. The criteria used to classify carotenoid-containing were that items must have had ≥ 0·1 mg per serving of either α-carotene, *β*-carotene, *β*-cryptoxanthin, lycopene, lutein and zeaxanthin (the most common forms of carotenoids in the human diet)^(35)^. Examples of carotenoid-containing foods and beverages included orange juice, tangerine fruit cups, avocadoes, string beans, tomatoes, green peppers, yams, spinach, eggs, sweet potato, salad and collards. Further details on food and beverage classifications can be found in Table 1.

**Table 1:** Decision criteria for coding bag check food and beverage items as healthy or unhealthy, and example food and beverage items*

Category	Decision criteria	
	Healthy	Unhealthy
Juice	100 % juice Documented with enough detail that researcher could look up nutrient information to determine the juice was 100 % juice Examples: orange juice, cranberry apple, cranberry, juicy juice, orange tangerine and pineapple	Not 100 % juice Non-descript juice (e.g. ‘juice’) Examples: apple juice and cranberry apple raspberry
Beverages	Unsweetened beverages and wine Examples: unsweetened tea, green tea, red muscadine wine, white wine and wine	Sweetened beverages and beer/liquor Examples: sweet tea, lemonades, energy drinks, soda, smoothie, beer and liquor
Bread	All bread with few additives Examples: breads, buns, wholegrain, ciabatta, rye, flatbreads, rolls and tortillas	Breads with additives and pastries Examples: cheese breads, croissants, pastries (e.g. muffins) and sugary cereals
Pasta/rice	All pasta and rice with few additives Examples: white and wholegrain	Pasta and rice with additives Examples: macaroni-and-cheese and ramen noodles
Protein	Lean meats (e.g. a 3 oz serving with less than 10 grams of fat, 4·5 grams or less of saturated fat, and less than 95 milligrams of cholesterol)^(30)^ Examples: beef lengua, fish, carne asada, tuna, shrimp, chicken wings and chicken thighs	Non-lean meats, non-descript meats and high-fat meats Examples: pork loin, steak, beef, any meat fried or referred to as a ‘plate’, sausage, bologna and turkey lunch meat
Snacks	Non-sugary and non-savoury snacks Examples: trailmix, plain popcorn and plain nuts	All sugary or savoury-flavoured snacks Examples: crackers, chips, seasoned nuts (e.g. dill pickle peanuts), candy, cakes, donuts and white cheddar popcorn
Produce	All forms of produce were considered healthy.	
Dairy products	Yogurt and low-fat milk	Ice cream products

* All items not to be consumed by an adult (e.g. infant formula) or non-edible (e.g. gasoline and laundry detergent) were not coded or included in the denominator of proportion scores.

To calculate the Healthy Purchases score, participants received a score of 1 if any healthy items were purchased and a score of 0 if no healthy items were purchased. To calculate the Healthy Purchases-Proportion score, all items in a purchase were scored as healthy (1) or not healthy (0) and summed, and the number of healthy purchases was divided by the total number of items purchased (healthy and unhealthy). Multi-component items (e.g. BLT sandwich) were coded as their individual components (e.g. bacon, lettuce, tomato and bread).

To calculate the Carotenoid-Containing Purchases score, participants received a score of 1 if any carotenoid-containing items were purchased and a score of 0 if no carotenoid-containing items were purchased. To calculate the Carotenoid-Containing Purchases-Proportion score, all items in a purchase were scored as a carotenoid-containing food (1) or non-carotenoid-containing foods (0), and carotenoid-containing foods were summed and then divided by the total number of items purchased (carotenoid and non-carotenoid containing foods). Multi-component items were coded as their individual components, similar to coding for the healthy proportion purchases.

### Analysis

Data analyses were conducted using SAS version 9.4 (SAS Institutes). Univariate statistics, including means and frequencies, were used to describe demographic variables. Chi-square tests and two-sample *t* tests were used to compare the demographics between those who purchased healthy/carotenoid items and those who did not. To examine associations between purchase of healthy items and carotenoid-containing items and VM®-assessed skin carotenoids, *t* tests and multiple linear regression models were computed, including covariates of sex, age, race and smoking status. These covariates were included because they could all be associated with FV intake and with SCS^(36–39)^. The four main independent variables considered were Healthy/Carotenoid-Containing Purchasing scores and Healthy/Carotenoid-Containing Purchases-Proportion scores. Data were collected for 1086 participants; however, due to missing data, sample sizes ranged from 966 to 967 in linear regression models. In an analysis of missing data, there were no significant differences between those missing and not missing data on the variables of interest (healthy/carotenoid-containing purchases and skin carotenoids). For bivariate analyses between proportion of healthy items and proportion of carotenoid-containing items, Pearson’s correlation coefficients were used, with Cohen’s criteria for the strength of association (0 < 0·3 = small; 0·3–0·5 = moderate; > 0·5 = large)^(40)^.

## Results

### Healthy and carotenoid-containing foods and beverages purchased

Table 1 shows examples of healthy foods purchased, including any produce purchased, lean meats, 100 % juices, plain popcorn and plain nuts, milk, and yogurt. Unhealthy items (the majority of items purchased) included fatty meats, non-100 % juices, crackers, chips, candy, cakes, and flavoured popcorn and peanuts with excessive added salt, sugar or fat.

The carotenoid-containing foods purchased included orange juice, cranberry apple juice, cranberry apple raspberry juice, pineapple juice, tangerine fruit cup, avocadoes, string beans, tomatoes, green peppers, yams, spinach, tomatoes and sweet potatoes.

### Participant characteristics

The characteristics of study participants are found in Table 2, for the overall sample, and by those purchasing healthy and carotenoid-containing food and beverage items. Most participants were male (55·1 %) and either Black/African American (60·0 %) or White (27·6 %). The mean participants’ age was 43·4 years (sd = 15·3). The mean VM®-assessed skin carotenoids was 236·4 (sd = 81·9). (Data not shown.)

**Table 2:** Characteristics of 1086* small food retailer store participants, overall and by number and percentage with healthy and carotenoid-containing food and beverage purchases

Characteristics	*n*	%	*n* with healthy purchases	%	*n* with carotenoid-containing purchases	%
Sex*						
Male	597	55·1	217	36·4	45	7·54
Female	479	44·2	173	36·1	37	7·72
Other	7	0·7	4	57·1	0	0
*P*-value**			0·937		0·909	
Race/ethnicity*						
Black/African American	643	60·0	200	31·1	38	5·91
White	296	27·6	133	44·9	30	10·1
Other	133	12·4	57	42·9	11	8·27
*P*-value			< 0·001		0·065	
Smoking*						
Yes	500	50·2	127	25·4	21	4·20
No	497	49·8	232	46·7	53	10·7
*P*-value			< 0·001		< 0·001	
Age*			With *v*. W/O healthy purchase		With *v*. W/O carotenoid-containing purchases	
Mean	43·4		45·2 *v*. 42·3		44·8 *v*. 43·3	
sd	15·3		15·3 *v*. 15·2		16·5 *v*. 15·2	
*P*-value			0·003		0·426	

* Individuals did not report demographics for the following characteristics: age (*n* 15), sex (*n* 3), race/ethnicity (*n* 14) and smoking (*n* 89).

** Other category was excluded.

### Associations between healthy items purchased, proportion of healthy items purchased and skin carotenoid scores

Table 3 shows that among the 395 participants who purchased a healthy item, mean VM®-assessed SCS was 246·6 (sd = 84·3), and among the 691 participants who did not purchase a healthy item, mean VM®-assessed SCS was 230·6 (sd = 80·0). This difference was statistically significant, *P* = 0·002 (Cohen’s D = 0·20). There was also a small, positive statistically significant correlation between proportion of healthy items purchased and VM®-assessed SCS (*r* = 0·077, *P* = 0·011). (Data not shown.)

**Table 3:** Mean Veggie Meter®-assessed skin carotenoid scores for those with and without healthy and carotenoid-containing purchases

Food and beverage items purchased	*n*	%	Mean Veggie Meter®-assessed skin carotenoid score	sd	*P*-value for difference between groups	Cohen’s D
Healthy purchases						
Yes	395	36·4	246·6	84·3	0·002	0·20
No	691	63·6	230·6	80·0		
Carotenoid-containing purchases						
Yes	83	7·6	260·2	92·2	0·006	0·32
No	1003	92·4	234·5	80·8		
Healthy purchases of non-carotenoid items						
Yes	315	31·4	243·2	81·7	0·020	0·16
No	688	68·6	230·5	80·0		

Table 4 shows that, when controlling for sex, age, race and smoking status, purchase of a healthy item was positively and statistically significantly associated with VM®-assessed SCS, such that individuals who purchased a healthy item had significantly higher VM®-assessed SCS compared to those who did not purchase a healthy item (estimate = 12·96; se = 5·31; *P* = 0·015).

**Table 4:** Adjusted linear regression analyses* for the association between healthy purchases, carotenoid-containing purchases and Veggie Meter®-assessed skin carotenoid scores among 1086 study participants. The outcome SCS had a mean 236 (82)**

Independent variable	B	se	*P*	Independent variable	B	se	*P*
*n* 967, R^2^ = 0·105				*n* 966, R^2^ = 0·103			
Healthy items purchased (yes *v*. no)	12·96	5·31	0·015	% Healthy items purchased	0·13	0·07	0·057
Age	−0·68	0·17	< 0·001	Age	−0·68	0·17	< 0·001
Sex (male *v*. female)	44·52	4·97	< 0·001	Sex (male *v*. female)	44·94	4·98	< 0·001
Race (Black *v*. White)	17·41	5·73	0·009	Race (Black *v*. White)	16·54	5·71	0·014
Race (Other *v*. White)	15·66	8·6		Race (Other *v*. White)	14·72	8·62	
Smoking (no *v*. yes)	19·24	5·12	< 0·001	Smoking (no *v*. yes)	19·97	5·1	< 0·001
*n* 967, R^2^ = 0·105				*n* 966, R^2^ = 0·109			
Carotenoid items purchased (yes *v*. no)	23·77	9·58	0·013	% Carotenoid items purchased	0·41	0·13	0·002
Age	−0·66	0·17	< 0·001	Age	−0·68	0·17	< 0·001
Sex (male *v*. female)	44·58	4·97	< 0·001	Sex (male *v*. female)	44·55	4·97	< 0·001
Race (Black *v*. White)	16·62	5·69	0·012	Race (Black *v*. White)	17·23	5·7	0·008
Race (Other *v*. White)	16·10	8·61		Race (Other *v*. White)	17·10	8·61	
Smoking (no *v*. yes)	20·15	5·06	< 0·001	Smoking (no *v*. yes)	20·50	5·04	< 0·001

SCS, skin carotenoid status.

* Data were collected for 1086 participants; however, due to missing data, sample sizes ranged from 966 to 967 in linear regression models.

** The models are adjusted for sex, age, race and smoking.

In adjusted linear regression analyses controlling for age, sex, race and smoking, % healthy items purchased was positively associated with VM®-assessed SCS, such that individuals who purchased 1 % more of healthy items had a higher VM®-assessed SCS (estimate = 0·13; se = 0·07; *P* = 0·057).

In the four models in Table 4, age was associated with SCS such that older individuals had lower VM®-assessed SCS. Sex was associated such that males had higher VM®-assessed SCS than females. Race was associated such that Black and other races had higher VM®-assessed SCS than White individuals. Smoking was associated with VM®-assessed SCS such that those who did not smoke had higher VM®-assessed SCS compared to those who did.

Table 3 also shows that purchase of healthy non-carotenoid containing items was also associated with higher VM®-assessed SCS such that those who purchased any healthy non-carotenoid containing items had a significantly (*P* = 0·020) higher SCS (243·2 (81·7) compared to those who did not (230·5 (80·0)).

### Associations between carotenoid-containing items purchased, proportion of carotenoid-containing items purchased and skin carotenoid scores

Table 3 shows that among the eighty-three participants who purchased a carotenoid-containing item, mean VM®-assessed SCS was 260·2 (sd = 92·2). Among the 1003 participants who did not purchase a carotenoid-containing item, mean VM® assessed SCS was 234·5 (sd = 80·8). The difference was statistically significant, *P* = 0·006 (Cohen’s D = 0·32). There was also a small, positive statistically significant correlation between proportion of carotenoid-containing items and VM®-assessed SCS (*r* = 0·096, *P* = 0·002). (Data not shown.)

Table 4 shows that, when controlling for sex, age, race and smoking status, purchase of a carotenoid-containing item was positively and statistically significantly associated with VM®-assessed SCS, such that individuals who purchased any carotenoid-containing items had significantly higher VM®-assessed SCS compared to those who did not purchase any carotenoid-containing items (estimate = 23·77; se = 9·58; *P* = 0·013).

In the adjusted linear regression model, % carotenoid-containing items purchased was positively and statistically significantly associated with VM®-assessed SCS, such that individuals who purchased 1 % more of carotenoid-containing items had a higher VM®-assessed SCS (estimate = 0·41, se = 0·13, *P* = 0·002).

## Discussion

In the current study, participants who purchased healthy and/or carotenoid-containing foods had significantly higher skin carotenoids than those who did not purchase any healthy or carotenoid-containing foods. A previous study suggests that FV purchases have the highest concordance between purchases and consumption^(41)^. Our findings suggest that healthy and carotenoid-containing purchases at small food stores are possibly indicative of carotenoid-rich FV intake due to the association with SCS.

The strengths of this study include large sample size and the use of an objective dietary measurement. Additionally, the study was conducted in an underserved area, where communities could benefit greatly from this research. Limitations include the use of a convenience sample from one state, which reduces generalisability. Participants were also not asked whether they were purchasing items for themselves or for others. For this reason, we do not know if and what proportion of food/beverages purchased were consumed by the participant. However, many items purchased from small stores are single-serve snacks or ready-to-eat foods^(42)^, increasing the likelihood that the participant was also the consumer of the food/beverage. A further limitation is that the determination of what constituted an ‘item’ was challenging given the varied range of foods purchased. Yet a more precise measure based on weight was not feasible in this study.

Finally, there are many ways to define healthy and unhealthy foods/beverages and the definitions chosen may be debated. Nevertheless, the use of three health and nutrition professionals to code healthy and unhealthy foods based on established nutrition-related guidelines adds increased credibility to the results.

### Conclusions

Customers who purchased healthier food and beverages from small food retailers had higher skin carotenoids. This suggests that customer purchases at small stores are indicative of dietary healthfulness as approximated using skin carotenoids. Further, these customers who purchased foods higher in carotenoids, and had higher skin carotenoid levels, did so without a specific intervention aimed at increasing skin carotenoid scores. More research is needed to understand additional factors that could improve the number of carotenoid-rich foods offered and the percentage of individuals who purchase carotenoid-rich foods at small food retailers. Lastly, the current study indicates that measuring both purchases of healthy and carotenoid-containing foods, as well as skin carotenoids, are both potentially feasible and effective tools in evaluations of similar public health nutrition interventions.
